# UV/Ozone Treatment of Polymer Surfaces to Enhance Cell Adhesion: The Mechanism and Guidelines for Optimization

**DOI:** 10.1021/acs.langmuir.5c03398

**Published:** 2025-10-22

**Authors:** Riko Kaizu, Seiichiro Takahashi, Kenichi Hirose, Kenji Hatakeyama, Glenn Villena Latag, Ayano Nomura, Hiroyuki Tahara, Tomohiro Hayashi

**Affiliations:** † Department of Materials Science and Engineering, School of Materials and Chemical Technology, 693022 Institute of Science Tokyo, Yokohama 226-8502, Japan; ‡ 88496 Ushio Inc., 6409 Motoishikawa-Cho, Aoba-Ku, Yokohama-Shi, Kanagawa-Ken 225-0004, Japan

## Abstract

Despite the widespread use of UV/ozone (UVO) treatments to improve cell adhesion and proliferation on polymer substrates, a complete understanding of the mechanism has not been achieved. This study investigates the effect of UVO treatment on the physicochemical properties of polymer surfaces (polystyrene and cyclo-olefin polymer) and protein adsorption, focusing on its impact on cell adhesion and the underlying mechanisms. UVO treatment with short time (1 or 2 min) significantly enhances cell adhesion, whereas treatment longer than 10 min results in poor adhesion. The treatment introduces oxygen-containing functional groups and increases wettability; however, the results indicate that wettability alone is not a determining factor for cell adhesion. Atomic force microscopy (AFM) imaging revealed nanoscale structural changes on treated surfaces, while enzyme-linked immunosorbent assay (ELISA) and quartz crystal microbalance with energy dissipation (QCM-D) analysis demonstrated that protein adsorption and denaturation are influenced by treatment duration. Additionally, the study observed the Vroman effect, showing that protein exchange on UVO-treated surfaces changes the composition of the protein layer. It was further suggested that on surfaces with short UVO treatment, fibronectin (FN) and vitronectin (VN) trapped on remaining hydrophobic areas serve as cell recognition sites, thus promoting adhesion. Overall, these findings reveal that UVO treatment duration influences protein adsorption on polymer surfaces and improves cell attachment, offering valuable insights for designing better tissue culture surfaces and enhancing material-cell interactions in biomedical contexts.

## Graphical abstract

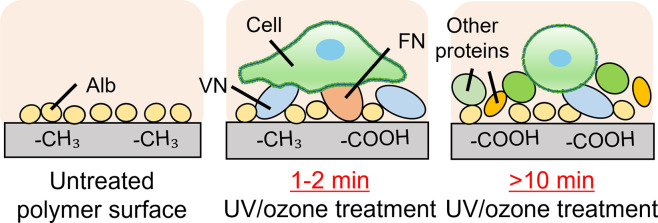

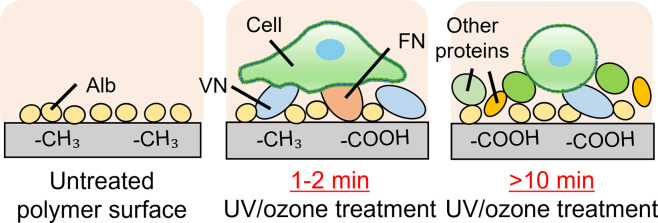

## Introduction

UV/ozone (UVO) or plasma treatments are widely used to improve cell adhesion and proliferation on polymer surfaces. The treatments of polystyrene (PS), which is the material of typical cell culture dishes, render the surface hydrophobic and significantly improve the adhesion of various cells such as Vero cells,^1^ mouse embryonic stem cells,^2,3^ human pluripotent stem cells,^4^ Chinese hamster ovary cells,^5,6^ and others.^7–17^ Several studies confirmed that the UVO treatment time is a critical factor, i.e., both insufficient and excessive UVO exposure on polymer surfaces led to the formation of undesirable surfaces for cell adhesion.^4,11,12^ However, the underlying mechanism has been a long-standing question because of the lack of understanding of the interfacial molecular processes, ranging from the changes in physicochemical properties of the surface to the distribution of cell adhesion sites.

Understanding the connection between the physicochemical properties of modified surfaces (wettability, functional groups, topography, etc.) and cellular responses (adhesion, proliferation, etc.) is essential for optimizing the cell-culturing substrates. The chemical composition of modified polymer surfaces were studied by using X-ray photoelectron spectroscopy (XPS),^2–6,9,13,15,16^ time-of-flight secondary ion mass spectrometry (ToF-SIMS),^4,11,12^ and Fourier-transform infrared spectroscopy (FT-IR).^1,8,17^ These studies have reported the increase in the formation of oxygen-containing functional groups, such as carbonyl and carboxyl groups.

Microscopic topographic changes of the polymer surface induced by UVO or plasma treatments and their effect on cell adhesion have also been investigated by atomic force microscopy (AFM).^5,6,13,14^ However, previous studies have reported inconsistent results regarding the effect of surface topography on cell adhesion. Several reports concluded that increases in surface roughness do not significantly affect cell adhesion because the nanostructures are much smaller than the cells.^5,13,15^ In contrast, some studies have suggested that roughened surfaces promote cell adhesion by increasing the interfacial area for proteins that scaffold the cells.^18,19^ Similarly, Tserepi et al. suggested that nanostructures formed on polymer surfaces by plasma treatment may promote cell adhesion.^14^

The study of cell adhesion is complicated because cells are affected not only by the physicochemical properties of the culture substrate but also by the complex culture environment, such as serum. This complexity makes it challenging to fully understand the interaction between the culture substrate and cells. Consequently, despite extensive studies on the effects of surface modification of culture substrates on cell adhesion, the underlying mechanism is not clearly understood. In cell culture in the presence of serum, proteins from the serum rapidly adsorb to the surface of the culturing substrate and interface the substrate and cells.

A previous study on protein adsorption following surface modification used XPS for semiquantitative evaluation of protein adsorption and reported an increase in total adsorbed proteins due to UVO treatment.^10^ Another study measured total protein adsorption using a bicinchoninic acid (BCA) assay and obtained similar results.^13^ Additionally, a study using quartz crystal microbalance with energy dissipation monitoring (QCM-D) measured the adsorption of single proteins, fibronectin (FN) and albumin (Alb), on PS. However, this study primarily compared the amount of protein adsorbed on UVO-treated PS and tantalum surfaces.^20^ The effects of UVO treatment of polymer surfaces on cell adhesion have also been investigated by precoating polymer surfaces with proteins such as FN,^15^ vitronectin (VN),^4,17^ γ-globulin,^17^ and tropoelastin,^8^ as well as by blocking with bovine serum albumin (BSA).^8^ However, no studies have comprehensively investigated the adsorption behavior of the proteins, including adsorbed amount, composition, and denaturation.

Cell adhesion proteins in serum, such as FN and VN, play an important role in cell adhesion.^21–24^ These cell adhesion proteins contain an intramolecular RGD sequence (arginine-glycine-aspartic acid), which specifically binds to integrins on the plasma membrane of the cell, promoting cell adhesion.^25,26^ Cell adhesion proteins adsorbed on the surface of the substrate are thought to undergo conformational changes, exposing their RGD sequences within the surface of the protein layer to form cell recognition sites.^27,28^ On the other hand, denatured cell adhesion proteins have been reported to reduce cell adhesion by causing a misalignment of integrin binding sites or inducing steric hindrance, although the overall effect of protein denaturation on cell adhesion has not been clearly determined.^29,30^ Additionaly, these studies indicated that RGD density affects cell adhesion efficiency, morphology, and cell spreading, with higher RGD surface density promoting greater cell spreading.^31^

The chemical modification of materials’ surfaces with RGD moieties has been studied to improve cell adhesion.^32,33^ This work reported that there are optimal RGD densities for cell adhesion, since too high RGD density can lead to surface crowding.^34^ The aforementioned studies clearly suggest that understanding the interaction between the material surfaces and cells requires a comprehensive investigation that includes the amount of adsorbed serum proteins on the material surfaces and their composition and denaturation.

Protein exchange on surfaces (also known as the Vroman effect) describes how proteins initially attached to a surface from a mixture are later replaced by proteins that arrive subsequently.^35^ In serum, Alb, which has low molecular weight and high abundance, adsorbs first but is later replaced by other proteins, affecting the composition of the protein layer. Observing this process is essential for a more detailed understanding of the adsorption behavior of serum proteins.

This study aims to comprehensively investigate the physicochemical characteristics of PS and cyclo-olefin polymer (COP) surfaces, examine the adsorption behavior of serum proteins, and elucidate how UVO treatment of polymer surfaces affects cell adhesion and its underlying mechanism. Mouse fibroblasts of the RGD-dependent type were cultured on polymer substrates and cell adhesion, including the cell densities and proliferation, were evaluated. XPS, water contact angle (WCA) measurements, and AFM were used to investigate atomic compositions, wettability, and topography of polymer surfaces, respectively. The adsorption behavior (adsorbed amount, denaturation, composition, and exchange process) of serum proteins was also investigated using enzyme-linked immunosorbent assay (ELISA) and QCM-D. A model of adsorbed serum proteins on polymer surfaces based on the Vroman effect was then constructed. The changes in the material surfaces induced by UVO treatment helped explain the mechanisms influencing protein-adsorption behavior and its effects on cell adhesion.

## Materials and Methods

### Substrate Preparation by Spin Coating

A 2% (w/w) PS solution was prepared by dissolving PS beads (Sigma-Aldrich, St. Louis, MO, USA) in toluene (FUJIFILM Wako Pure Chemical Corporation, Osaka, Japan). COP beads (Zeonex 690R, Zeon Corporation, Tokyo, Japan) were added to cyclooctane (Fujifilm Wako Pure Chemicals) to achieve a concentration of 2% (w/w), and the resulting mixture was heated at 60 °C overnight to ensure complete dissolution. PS- and COP-coated substrates were fabricated by spin-coating 50 μL of each solution onto micro cover glass (15 mm diameter, Matsunami Glass Ind., Ltd., Osaka, Japan) at 2000 rpm using a spin-coating machine (ACE-200, HiSOL, ink., Tokyo, Japan).

### UV/Ozone (UVO) Treatment

UVO treatment was performed by exposing the polymer-coated substrates in a Mini-Excimer irradiation unit (172 nm, Ushio Inc., Tokyo, Japan) at a constant distance of 2 mm from the source. The intensity of Xenon excimer lamp used was 5.21 mW/cm^2^. The polymer-coated substrates underwent UVO treatment for varying irradiation times ranging from 0 to 16 min. Although UVO-treated polymer surfaces start to become more hydrophobic immediately after treatment, it has been reported that the treated polymer surfaces retain higher wettability than the original untreated surfaces even after 28 days.^36^ Therefore, all characterizations were conducted after storing the samples in the dark under ambient air at room temperature for 24 h following UVO treatment to reduce the effect of wettability changes on the results.

### Cell Culture

Mouse BALB 3T3 A31-1-1 clone cells (JCRB Cell Bank in the National Institute of Hygienic Sciences, Tokyo, Japan) were used to evaluate the effect of UVO treatment on PS and COP surfaces regarding cell adhesion. For the cell culture experiments, the cells were grown in a tissue culture PS flask for 3 days in Dulbecco’s Modified Eagle Medium (DMEM) (High Glucose, FUJIFILM Wako Pure Chemical Corporation, Osaka, Japan), supplemented with 10 w/w% fetal bovine serum (FBS) (Gibco; ThermoFisher Scientific, Waltham, MA, USA). Following the culture period, the cells were harvested using 0.25% trypsin-EDTA (Gibco; ThermoFisher Scientific, Waltham, MA, USA). A polymer-coated cover glass was placed in each well of a 24-well plate (Costar 3513, Corning Incorporated, Corning, NY, USA), and the cells were seeded onto the polymer-coated substrates at a density of 6000 cells per well and incubated for 24 h. After incubation, the well surfaces were rinsed with phosphate-buffered saline (PBS) (Takara Bio Inc., Shiga, Japan), and the cells were fixed with 4% paraformaldehyde (Sigma-Aldrich, St. Louis, MO, USA) in PBS and then permeabilized using 0.2 v/v% Tween-20 (Sigma-Aldrich, St. Louis, MO, USA). Images were acquired using an inverted microscope (Olympus CKX41, Olympus, Tokyo, Japan). The cell densities, circularities of adhered cells, and area per cell were quantified using ImageJ.^37^

### X-ray Photoelectron Spectroscopy (XPS)

X-ray photoelectron spectroscopy (XPS) (Quantera II, ULVAC-PHI, INC., Kanagawa, Japan) was employed to analyze the elemental and functional composition of polymer surfaces. The base pressure in the XPS analysis chamber was approximately 1 × 10^–7^ Pa. The samples were excited with monochromatic Al Kα radiation of 1486.6 eV over an area of 7850 μm^2^. Photoelectrons were detected with a hemispherical analyzer positioned at a takeoff angle of 45°. XPS survey spectra were collected at a pass energy of 224.0 eV using an energy step of 0.8 eV, while high-resolution C 1s spectra were recorded at a pass energy of 55.0 eV using an energy step of 0.1 eV. The surface elemental composition was calculated based on the peak areas obtained from narrow scan spectra, which were fit using a Gauss-Lorentz function after the Shirley background was subtracted using MultiPak v9.8 software.

### Water Contact Angle (WCA) Measurement

Static WCAs of untreated and UVO-treated polymer surfaces were measured using the θ/2 method on a contact-angle meter (DMe-201, Kyowa Interface Science Co., Ltd., Saitama, Japan) at room temperature. All droplets had a volume of 18 μL. The data were analyzed using FAMAS v3.5.5 software (Kyowa Interface Science Co., Ltd., Saitama, Japan).

### Surface Topographic Measurements in Air

The topography of polymer surfaces was measured using an AFM (L-trace II, Hitachi High-Tech Science Corporation, Tokyo, Japan) at room temperature in dynamic force mode. The cantilever consisted of silicon (SI-DF40, spring constant: 40 N/m, tip radius: ≤10 nm, back side: Al coating, Hitachi High-Tech Science Corporation, Tokyo, Japan). The scan speed was set to 1.0 line/Hz, and images were captured over an area of 5 × 5 μm^2^. The number of sampling points was 512 × 512 in both cases. Images were analyzed with Gwyddion software.

### Surface Topographic Measurements in PBS

The topographic images of polymer surfaces in PBS (Sigma-Aldrich, St. Louis, MO, USA) were obtained using a commercial AFM system equipped with a liquid cell (MPF-3D Infinity, Oxford Instruments, U.K.). Measurements were performed at room temperature (25 °C) in fast force mapping mode. The cantilever was constructed from silicon (BioLever mini, BL-AC40TS-C2, nominal spring constant: 0.1 N/m, tip radius: 8 nm, Olympus, Tokyo, Japan). The scan rate was set to approximately 0.06 Hz, and images were captured over an area of 5 × 5 μm^2^ with 256 × 256 pixels.

### Enzyme-Linked Immunosorbent Assay (ELISA)

Polymer-coated cover glasses were attached to 96 bottomless well plates (CSTEC CO., Ltd., Kyoto Japan). The wells were coated with a 10 w/w% FBS solution in DMEM, diluted 500-fold in PBS (Takara Bio Inc., Shiga, Japan), and incubated at 37 °C for 1 h. Afterward, the wells were emptied and washed three times with 0.05% Tween-20 (FUJIFILM Wako Pure Chemical Corporation, Osaka, Japan) in PBS. The wells were then incubated with PBS containing 0.5% (w/v) BSA (FUJIFILM Wako Pure Chemical Corporation, Osaka, Japan), followed by additional washing. Subsequently, 100 μL of FN monoclonal antibody (A22) (ThermoFisher Scientific, Waltham, MA, USA), diluted 10,000 times in PBS, was added to each well, incubated at 4 °C overnight, and then washed again. Peroxidase-conjugated antimouse IgG secondary antibody (Jackson ImmunoResearch Inc., West Grove, PA, USA), diluted 5000 times in PBS, was added (100 μL per well). After a final incubation at 37 °C for 1 h, the wells were washed, and 100 μL of ELISA POD Substrate TMB kit (Nacalai Tesque, Inc., Kyoto, Japan) was added to each well to develop color for 15 min. Coloration was stopped using 100 μL of 1N sulfuric acid (FUJIFILM Wako Pure Chemical Corporation, Osaka, Japan), and absorbance was measured at 450 nm using a microplate reader (SH-1300lab, Hitachi High-Tech Science Corporation, Tokyo, Japan). The analysis of adsorbed VN was performed using the same method as for FN, except that the wells were coated with a 10 w/w% FBS solution in DMEM, diluted 300 times in PBS, and incubated at 37 °C for 1 h. Additionally, VN monoclonal antibody (HV23) (ThermoFisher Scientific, Waltham, MA, USA) was diluted 25,000 times in PBS.

### Quartz Crystal Microbalance with Energy Dissipation Monitoring (QCM-D) Measurements

Au-coated QCM-D sensors with a 5 MHz resonance frequency (Biolin Scientific AB, Gothenburg, Sweden) were cleaned using UVO treatment for 10 min, then immersed in a mixture of Milli-Q water, 25% ammonia (FUJIFILM Wako Pure Chemical, Osaka, Japan), and 30% hydrogen peroxide (FUJIFILM Wako Pure Chemical, Osaka, Japan) at 70 °C for 15 min. Afterward, the sensors were rinsed with Milli-Q water, dried with nitrogen gas, and subjected to a second UVO treatment for another 10 min. Polymers such as PS or COP were coated onto the QCM-D sensors via the same spin coating method used for cover glasses. The coated sensors were placed in the flow modules of the commercial QCM-D system (Biolin Scientific AB, Gothenburg, Sweden), with solutions injected using a peristaltic pump (Minato, Tokyo, Japan). Frequency and dissipation shifts were recorded during three steps: (1) establishing a baseline in DMEM, (2) introducing protein solutions in DMEM—this point marked as time = 0—and monitoring until signals stabilized, and (3) rinsing with DMEM until saturation. Serum adsorbed biomolecules were measured with 10 w/w% FBS in DMEM. Single protein adsorption of Alb (Sigma-Aldrich, St. Louis, MO, USA) was tested with 1 mg/mL Alb in DMEM. For sequential adsorption of Alb and FN, after baseline calibration in DMEM, 1 mg/mL Alb solution in DMEM was injected for 5 min, then rinsed with DMEM. Next, 0.1 mg/mL FN (Sigma-Aldrich, St. Louis, MO, USA) in DMEM was injected for 5 min until saturation. Sequential adsorption of Alb and FBS was similarly measured: 1 mg/mL Alb solution in DMEM followed by 10 w/w% FBS.

According to the classical mass loading theory proposed by Sauerbrey, the decrease in frequency observed in QCM-D measurements is directly proportional to the mass of particles adsorbed onto the surface, a phenomenon known as inertial loading. This relationship is expressed by eq 1

1 $$\Delta m=-C\frac{\Delta f}{n}$$

where *Δm, C, Δf,* and *n* represent the mass of the particles attached to the sensor surface, the mass sensitivity constant—an inherent characteristic of the quartz crystal, the shift in resonant frequency, and the overtone (harmonic) number, respectively. For an AT-cut quartz crystal operating at 5 MHz, the value of *C* is 17.7 ng/(cm^2^·Hz).

## Results and Discussion

### Changes in Adhered Cell Density after UVO Treatment

Figure 1 displays the cell densities after 24 h of cell culture on polymer surfaces treated with UVO at various exposure times. Although the UVO treatment drastically enhanced the cell adhesion, the longer treatment duration resulted in a decrease in the cell density. The highest cell density on COP and PS was acquired with UVO treatment for 1 and 2 min, respectively. Our results are consistent with the previous studies showing that short-time UVO treatment of polymer surfaces maximizes the density of the adhered cells, while long-time UVO treatment results in a reduced density of the adhered cells.^2,6,11,12^

**Figure 1:**
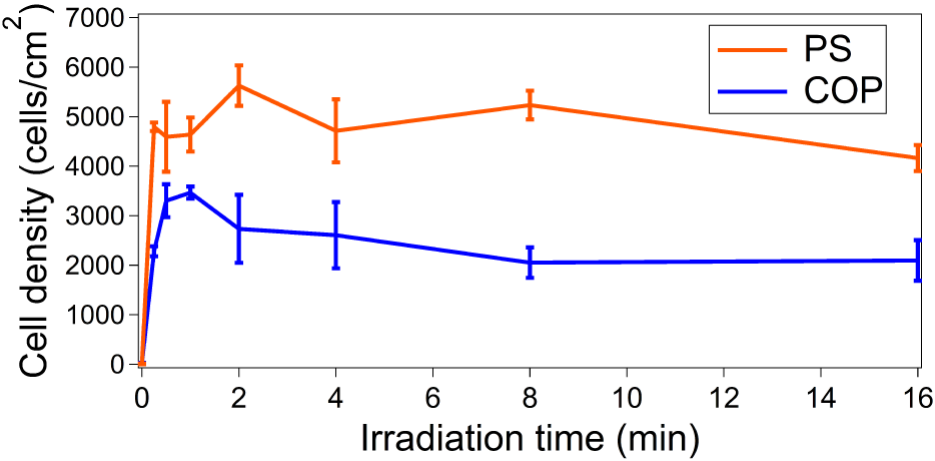
Densities of cells adhering to PS and COP after UVO treatments with varying irradiation times. Error bars indicate standard deviations (*N* = 3).

Figure 2 shows the optical microscope images of cells on polymer surfaces subjected to UVO treatment with various duration. As previously discussed, the enhancement in cell adhesion following the treatment was clearly evident. The treatment significantly influences not only cell density but also the morphologies of the cells. To evaluate the morphology of adhered cells, circularity and area per cell were calculated using images obtained through optical microscopy, as shown in Figure 3. Cells on polymer surfaces with short-duration UVO treatment demonstrate lower circularity and a larger adhesion area per cell compared to those on surfaces with longer treatment. In contrast, cells that adhered to surfaces with extended treatment exhibit a rounded morphology similar to that of suspended cells, accompanied by smaller adhesion areas.

**Figure 2:**
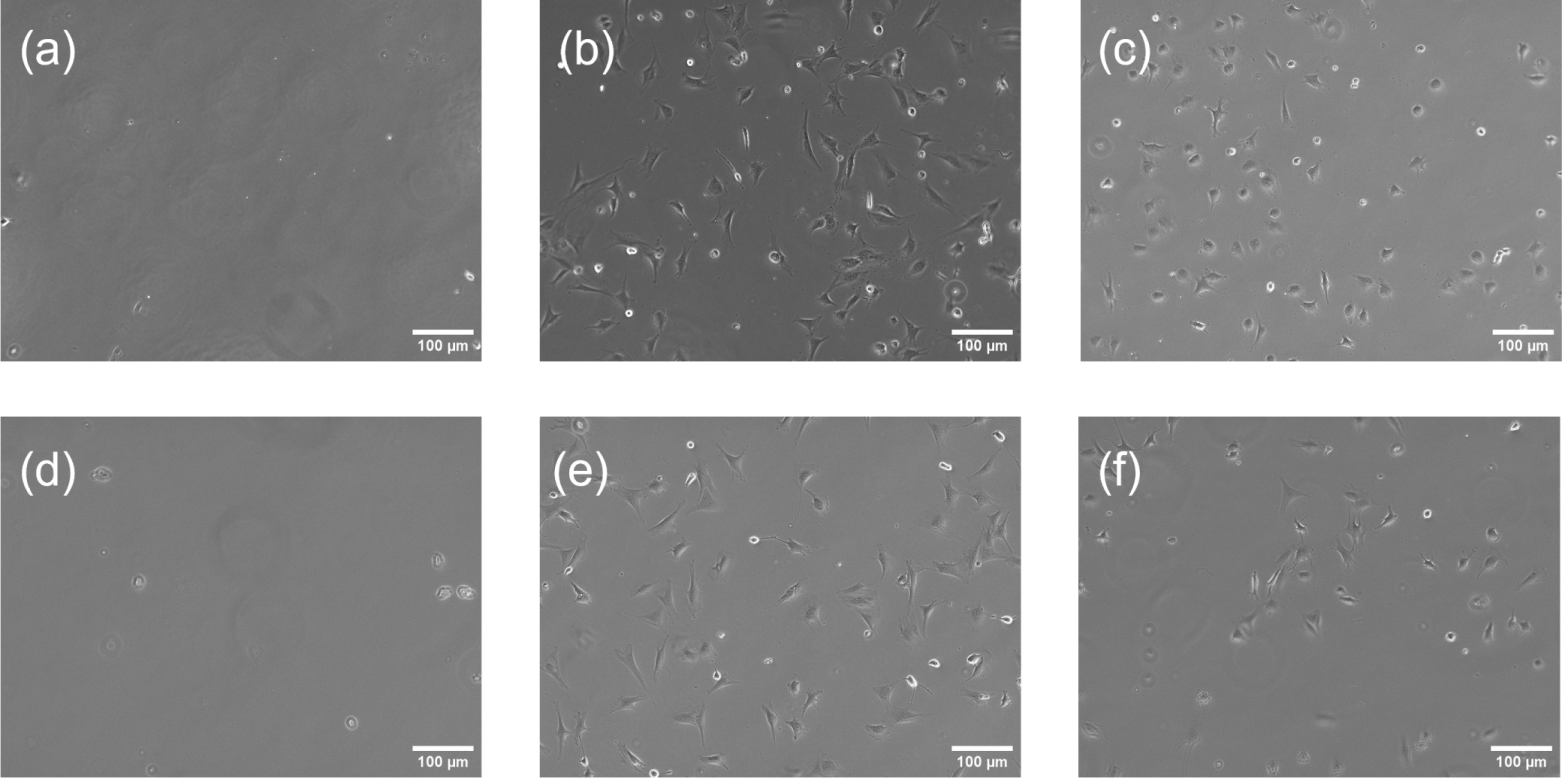
Optical microscope images of cells on COP after different durations of UVO treatment: (a) no treatment, (b) 1 min, (c) 16 min; and on PS: (d) no treatment, (e) 2 min, (f) 16 min.

**Figure 3:**
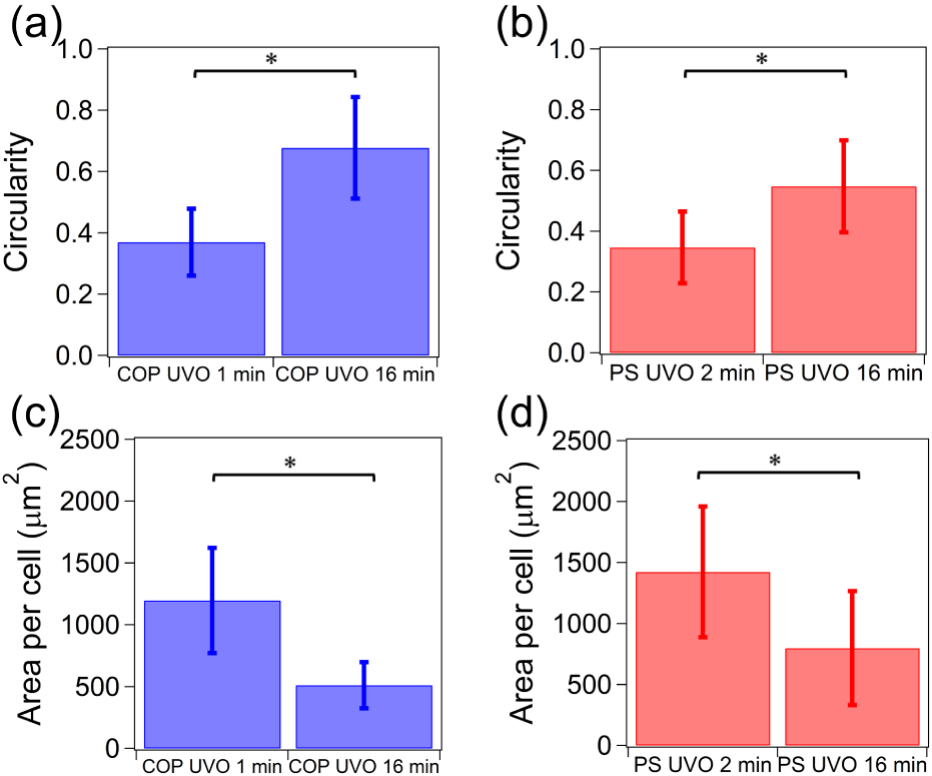
Circularity of cells cultured on (a) COP and (b) PS. Circularity measures how closely a two-dimensional shape resembles a perfect circle, using the formula (4π × area)/(perimeter^2^). A perfect circle has a circularity value of 1, while shapes that are less circular will have values less than 1. Area per cell cultured on (c) COP and (d) PS. Error bars denote standard deviation (*N* = 60), and asterisks (*) denote statistical significance (*p* < 0.05).

### Changes in Surface Functional Groups

Our XPS measurements revealed the chemical changes of the polymer surfaces induced by UVO treatment. The spectra were deconvoluted into four distinct components assigned to C–C/C–H, C–O, C=O and O–C=O using Gauss-Lorentz functions (Figure 4). In addition to these components, PS was fitted with two additional components, a π–π* of the benzene ring and a CO_3_ peak (assigned to benzene ring after UVO treatment). A summary of the peak fitting is summarized in Tables 1 and 2. These XPS results demonstrated that UVO treatment induced the formation of oxygen-containing functional groups, such as carbonyl, ether, and carboxyl, on the PS and COP surfaces. Our results are consistent with previous studies showing that UVO treatment introduces oxygen-containing functional groups on polymer surfaces.^1–3,5,9,11,12,38^ Carboxyl and hydroxyl groups have been reported as functional groups that may affect cell adhesion.^6,39–41^ It was also reported that excessive carboxyl group or excessive surface oxygen negatively impacts cell adhesion and proliferation.^2,5^ The results here suggest that cell adhesion is affected not only by surface functional groups.

**Figure 4:**
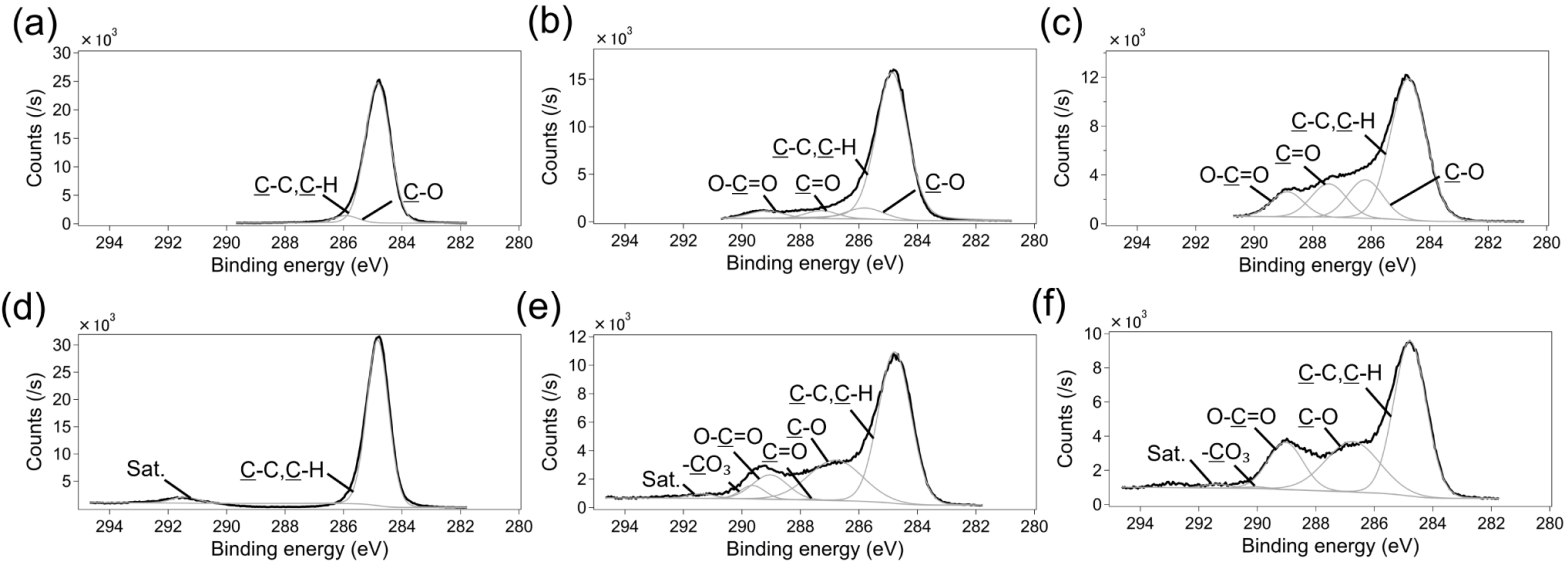
XPS spectra in the C 1s region of COP after different durations of UVO treatment: (a) no treatment, (b) 1 min, (c) 16 min; and PS: (d) no treatment, (e) 2 min, and (f) 16 min.

**Table 1:** Atomic Compositions of COP Surfaces Determined by XPS

		Peak area (%)		
Component	Position (eV)	No treatment	UVO 1 min	UVO 16 min
C–C, C–H	284.8	95.0	83.1	66.0
C–O	286.0	5.0	6.8	12.6
C=O	287.7	0.0	5.5	11.8
O–C=O	289.0	0.0	4.6	9.6

**Table 2:** Atomic Compositions of PS Surfaces Determined by XPS

		Peak area (%)		
Component	Position (eV)	No treatment	UVO 2 min	UVO 16 min
C–C, C–H	284.8	95.9	57.2	52.5
C–O	286.7	0.0	26.7	28.6
C=O	287.3	0.0	0.9	0.0
O–C=O	289.0	0.0	11.6	17.6
–CO_3_	289.6	0.0	3.3	0.2
Sat.	291.4	4.0	0.3	1.2

### Water Contact Angles (WCA)

The static WCAs of polymer surfaces treated with UVO are shown in Table 3. The WCA decreases monotonically with increasing UVO treatment time, due to the increase of oxygen-containing functional groups.^2,3,5,13,15^ Hydrophilic surfaces are generally believed to promote cell adhesion and spreading,^6,42–44^ and some reports suggest that surfaces with moderate hydrophilicity are desirable for cell adhesion.^45–48^ However, other research indicates that even though the hydrophilicity of UVO-treated surfaces diminishes over time, cell adhesion and the presence of functional groups remain stable from immediately after treatment.^14,17^ This suggests that wettability alone is not a decisive determinant of cell adhesion induced by UVO treatment. Our findings show that short-time UVO treatment, which maximizes cell adhesion, results in a broad WCA range—from 20° for PS to 60° for COP—further indicating that UVO’s influence on cell adhesion is not solely related to wettability.

**Table 3:** Water Contact Angles (WCAs) of COP and PS^a^

	WCA (24 h after UVO treatment) (°)
COP no treatment	97.5 (1.4)
COP UVO 1 min	60.6 (1.0)
COP UVO 16 min	13.9 (2.4)
PS no treatment	88.5 (3.5)
PS UVO 2 min	23.1 (3.3)
PS UVO 16 min	18.9 (10.9)

a Numbers in parentheses are standard deviations (*N* = 4).

### Changes in Surface Topography in Air and PBS

Figure 5 shows AFM topographic images of polymer surfaces at various UVO treatment durations. UVO treatment changes the topography of the polymer surfaces and increases the RMS surface roughness. The larger surface roughness observed in UVO-treated surfaces in liquid, compared to air, is likely due to hydrophilic polymer chains swelling and aggregating in the liquid. Previous research has shown that UVO or plasma treatments cause only minor surface roughness increases—usually a few to tens of nanometers—which are generally too small to influence cell adhesion, although nanostructured substrates can sometimes enhance adhesion.^5,13–15,18,19,49–51^ Furthermore, the nanostructures formed during UVO treatment caused only minimal increases in surface area (Table S1). While the exact mechanism of island-like structure formation of the UVO-treated PS surface in PBS remains unclear, XPS analysis indicated an increase in hydrophilic groups after 16 min of treatment compared to COP. The swelling and aggregation of hydrophilic polymer chains in PBS may have contributed to the development of these island-like features.

**Figure 5:**
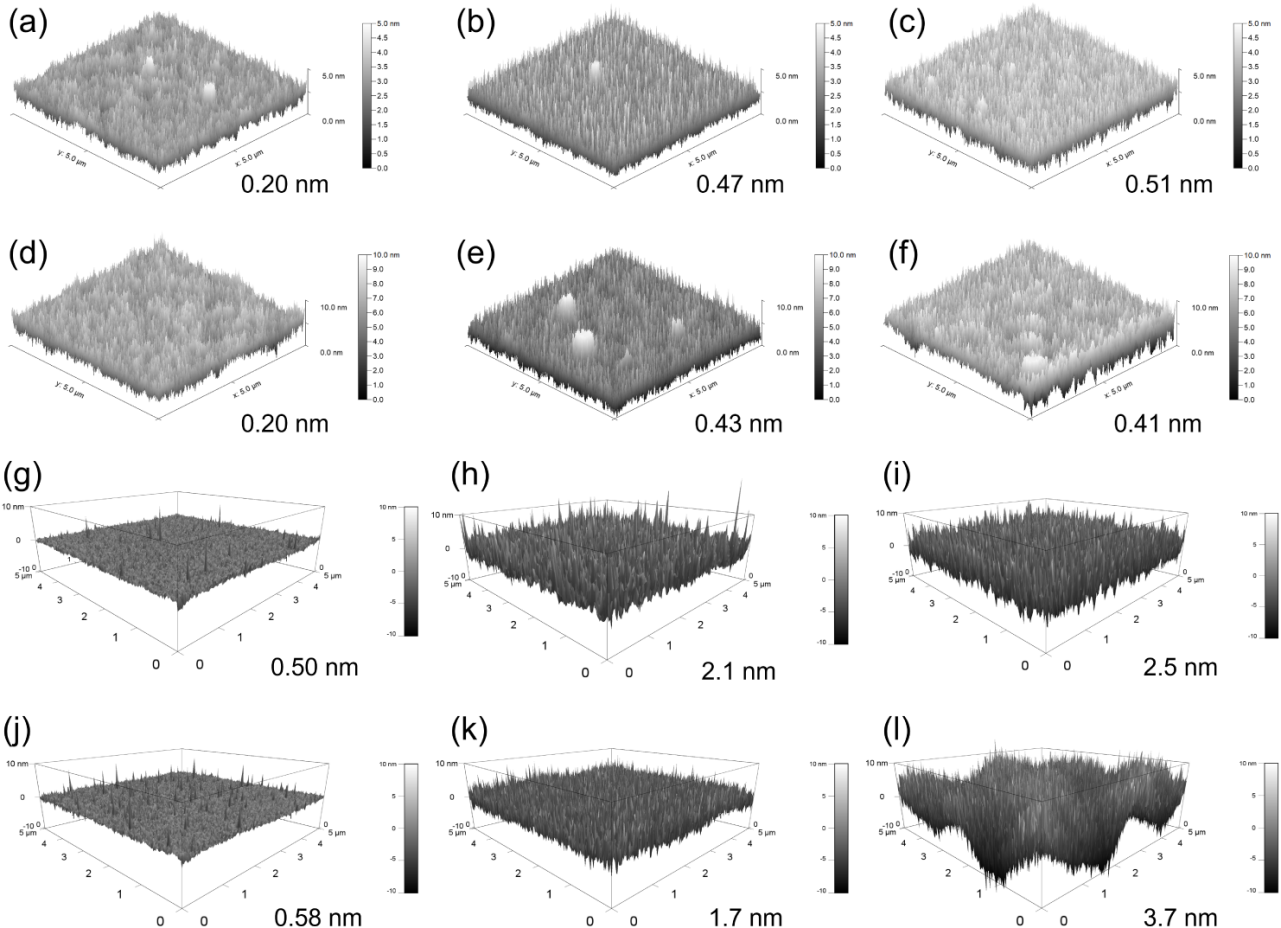
AFM images of COP in air after different UVO treatment durations: (a) no treatment, (b) 1 min, (c) 16 min; and PS: (d) no treatment, (e) 2 min, (f) 16 min. AFM images of COP in PBS after different UVO treatment durations: (g) no treatment, (h) 1 min, (i) 16 min; and PS: (j) no treatment, (k) 2 min, (l) 16 min. The values represent the RMS surface roughness.

In fast force mapping mode measurements in PBS, Young’s modulus was concurrently measured and analyzed using the Hertz contact model, as shown in Table 4. Results indicated that the surfaces of both polymers became increasingly softer with longer UVO treatment times. The reduction in Young’s modulus due to UVO treatment is attributed to the formation of hydrophilic groups on the polymer surface, which then swell in water. Surface roughness (related to Young’s modulus) in PBS increased, while actual Young’s modulus decreased, as a function of UVO exposure time. Consequently, we deduce that changes in surface roughness and Young’s modulus do not account for the improved cell adhesion observed at 1–2 min of UVO treatment.

**Table 4:** Average Values of Young’s Modulus of COP and PS^a^

	Young’s modulus (MPa)
COP no treatment	1432 (291)
COP UVO 1 min	23.01 (14.67)
COP UVO 16 min	10.81 (4.58)
PS no treatment	1502 (315)
PS UVO 2 min	90.92 (41.47)
PS UVO 16 min	22.58 (7.91)

a Numbers in parentheses are standard deviations (*N* = 10).

### Density of Fibronectin and Vitronectin Depending on UVO Treatment Time

In serum-containing culture, proteins like FN and VN that adsorb to the surface facilitate cell adhesion via their RGD–integrin interactions. To evaluate this, we analyzed FN and VN adsorption on UVO-treated polymer surfaces through ELISA (Figure 6a,b).

**Figure 6:**
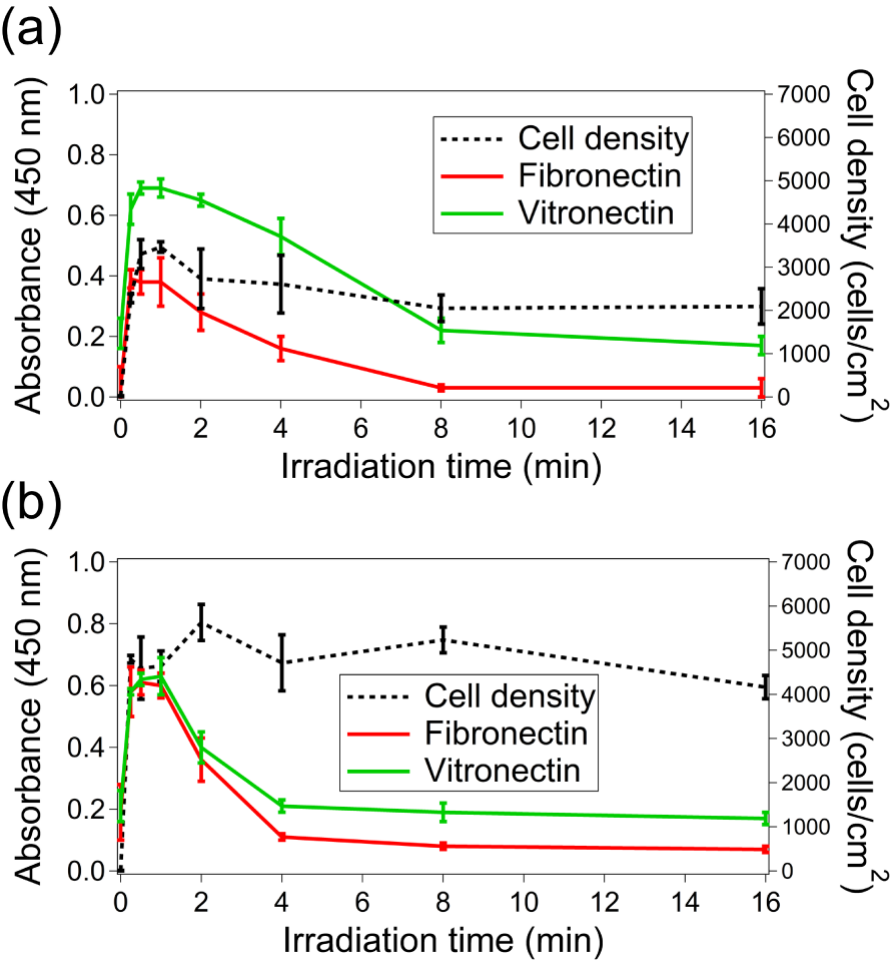
Amounts of adsorbed FN and VN (ELISA absorbance) on UVO-treated (a) COP and (b) PS substrates with different treatment times. The right *y* axes represent the density of the adhered cells (same data shown in Figure 1). Error bars denote standard deviation (*N* = 4).

Our findings show that short UVO treatments enhance FN and VN presence in serum adsorbed on the polymer surface or promote their accumulation on the protein layer’s surface. Conversely, longer UVO treatments of 8 to 16 min do not increase FN and VN enrichment. These observations align with changes in cell densities related to UVO treatment duration. Several studies indicate that RGD density influences cell morphology and adhesion,^31,34^ consistent with the observation that cells on surfaces with short UVO treatments exhibit lower circularity and a larger adhesion area per cell compared to those on surfaces with longer treatments (Figure 3).

### Protein Adsorption on Polymer Surfaces

Figure 7 a,b depicts the adsorption of serum biomolecules onto the QCM sensor coated with polymer films, showing the frequency shift (Δ*f*) over time. In QCM-D measurements, Δ*f* indicates the surface’s mass density, assuming a uniformly stiff (Sauerbrey) film, as illustrated in Figure 7c,d. Additional insights into the rigidity of the remaining layer can be obtained from the Δ*D*(last)/Δ*f*(last) ratios shown in Figure 7 e,f.

**Figure 7:**
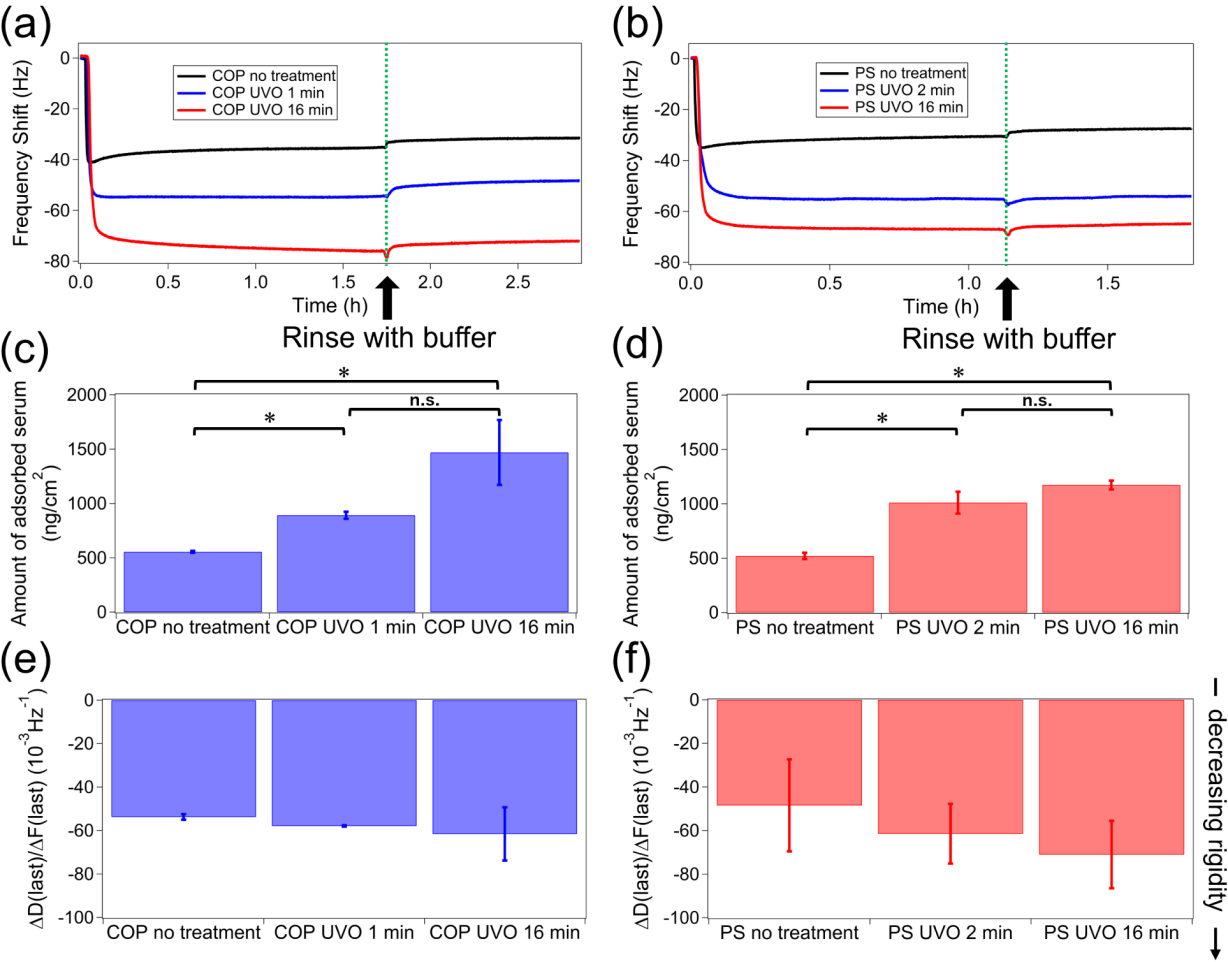
Kinetics of serum adsorption of biomolecules on (a) COP and (b) PS after different UVO treatment times. Saturated amounts of the adsorbed biomolecules on (c) COP and (d) PS, and viscoelasticity of adsorbed layer on (e) COP and (f) PS, are also shown for different UVO treatment times. Error bars denote standard deviation (*N* = 3), and asterisks (*) denote statistical significance (*p* < 0.05). The fifth overtone was used in these measurements.

The amount of serum adsorbed on the polymer surfaces increased with UVO treatment time. Although UVO induced nanostructure formation, the surface area increase was minimal (only a few percent), suggesting that surface area alone does not account for the enhanced adsorption. Instead, chemical changes, such as the generation of surface charges and functional groups caused by UVO, are more likely responsible for the increased serum binding, rather than topographical alterations. Additionally, the rigidity of the adsorbed layer decreased in tandem. Since protein mass includes hydration water, denaturation releases water molecules, reducing mass and increasing rigidity.^52^ The higher rigidity of serum on untreated surfaces indicates more significant denaturation compared to UVO-treated ones. The time profile of the Δ*f* reveals that when serum is introduced to the untreated polymer surface, Δ*f* profile shows a large negative shift upon serum adsorption to untreated surfaces, followed by a slight positive shift, implying rapid denaturation of adsorbed proteins, except Alb (discussed later). Generally, proteins on hydrophobic surfaces are more prone to denaturation,^53–57^ but UVO treatment, which increases hydrophilic groups, appears to prevent this. Interestingly, despite a decline in cell densities, FN, and VN after prolonged UVO treatment, the serum adsorbed more, indicating that the polymer surface’s chemical and structural changes influence the protein composition of the adsorbed serum.

To understand how UVO-treated polymer surfaces affect the rigidity of proteins during adsorption, plots of the energy dissipation signal (Δ*D*) against frequency shift were constructed (Figure 8). A steeper slope in these plots suggests a more dissipative (and therefore less rigid) material, while a shallower slope indicates greater rigidity.

**Figure 8:**
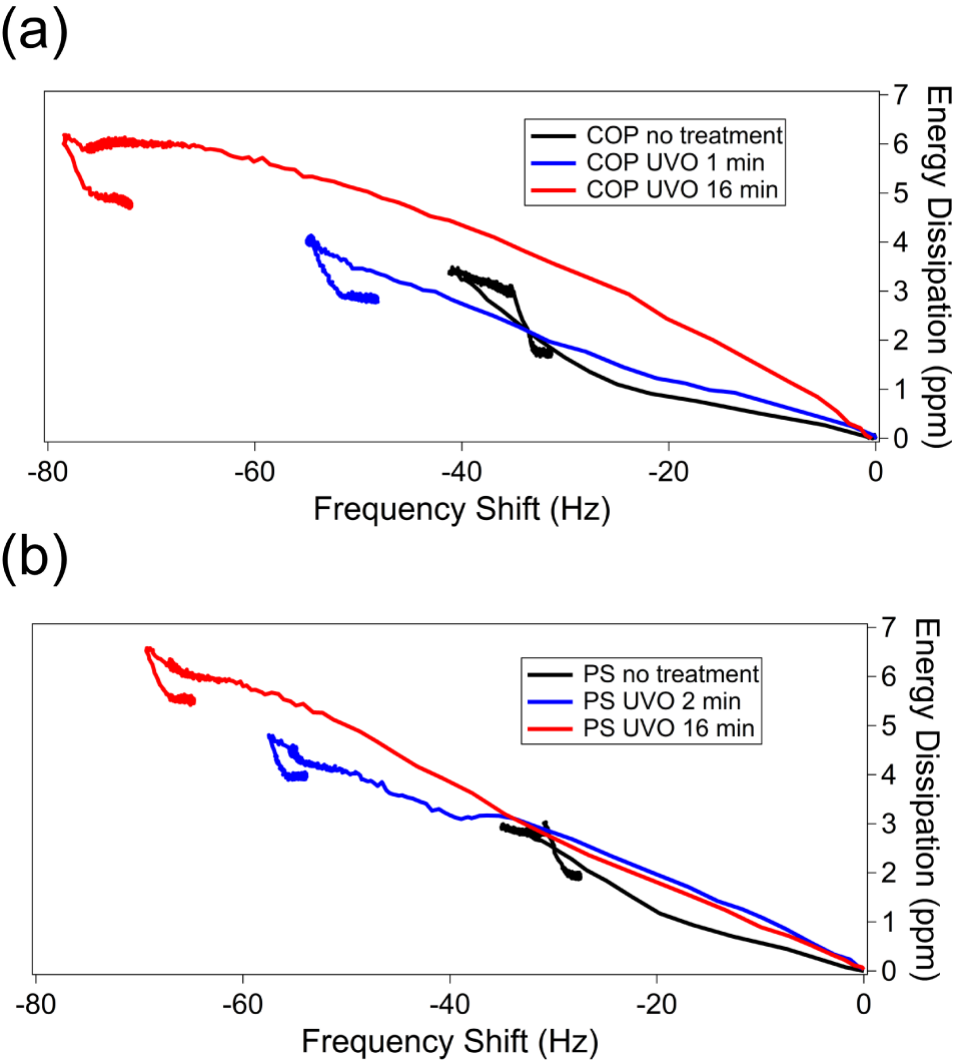
Δ*D* – Δ*f* plot showing the time progression of serum adsorption on (a) COP and (b) PS. The fifth overtone was used in these measurements.

Serum proteins initially adsorb onto the untreated polymer surface mainly through hydrophobic interactions, creating a strong protein layer that adheres firmly to the hydrophobic surface. As adsorption progresses, a steeper slope indicates that a softer, weaker protein layer forms, with proteins binding less tightly to each other. Conversely, on UVO-treated polymer surfaces, the adsorption shows a relatively consistent slope, indicating that serum proteins form soft, loosely bound layers less prone to denaturation. This results in a final layered structure that is less rigid. The Alb adsorption process was conducted in the same manner as for serum, as depicted in Figure 9.

**Figure 9:**
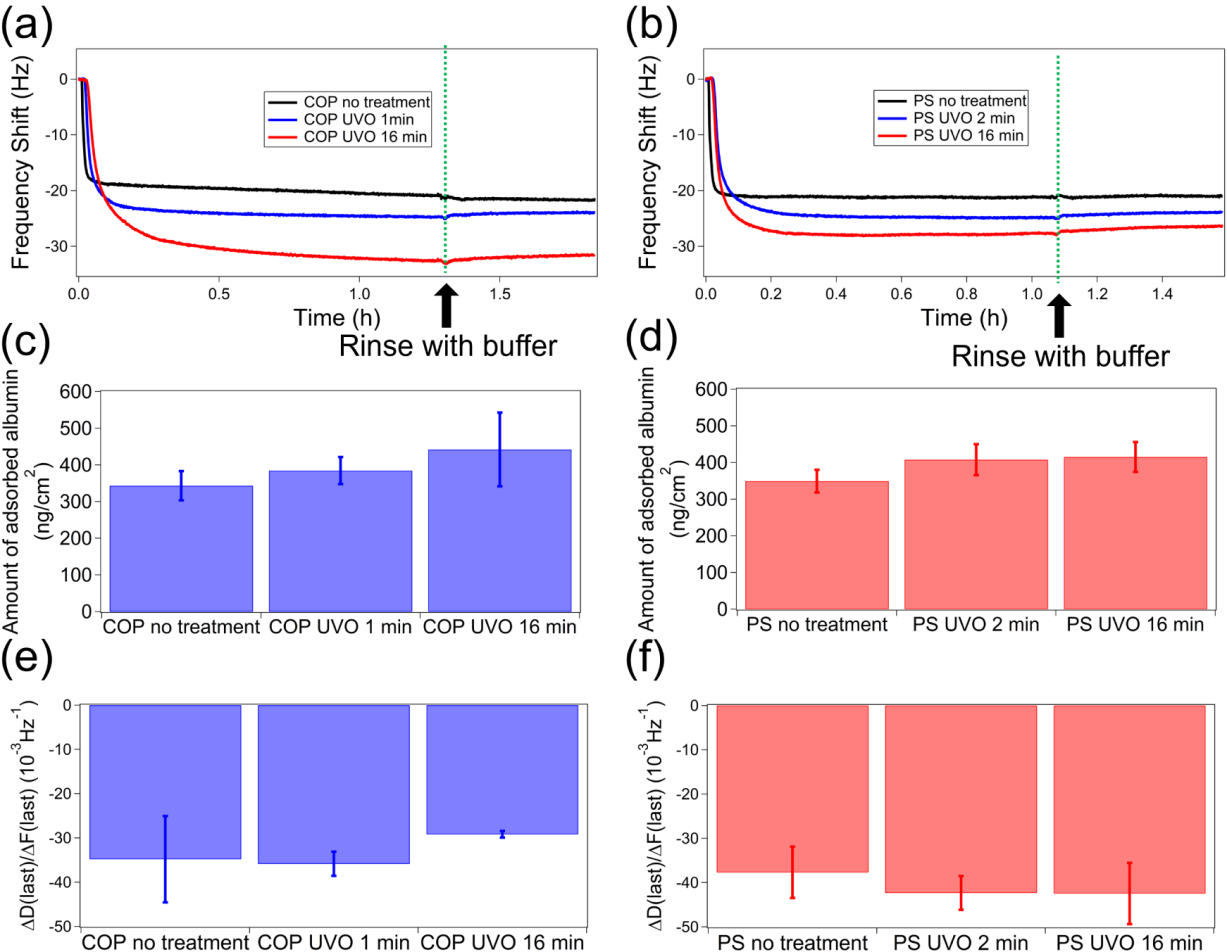
Kinetics of adsorption of Alb onto (a) COP and (b) PS after different UVO treatment times. Saturated amounts of Alb on (c) COP and (d) PS, and viscoelasticity of adsorbed layer on (e) COP and (f) PS, are also shown for different UVO treatment times. Error bars denote standard deviation (*N* = 3). The fifth overtone was used in these measurements.

A more detailed investigation into the behavior of adsorbed serum proteins focused on Alb, the most prevalent protein in serum. Although the amount of Alb adsorbed increased similarly to the serum as a whole, there were no notable differences in either Alb adsorption levels or its viscoelasticity across various treated surfaces. This suggests that the serum protein composition changes caused by UVO treatment are influenced more by proteins other than Alb. Moreover, the time profile of Δ*f* revealed that when Alb was injected onto untreated polymer surfaces, the typical large negative shift, followed by a slight positive shift seen with serum (Figure 7a,b), was absent. This indicates that the quick denaturation of proteins on untreated surfaces, observed with serum, is mainly due to serum proteins other than Alb.

The Δ*D* – Δ*f* plots were utilized to analyze the viscoelastic properties of the adsorbed Alb on each surface (see Figure 10). While there was no notable difference in the final stiffness of the adsorbed Alb layer (refer to Figure 9c,d), the initial rigidity during adsorption varied depending on the UVO treatment of the polymer surface. The curve shape on the untreated surface shifted from a gentle slope to a steeper one, suggesting that the initially adsorbed Alb layer forms a rigid structure, likely due to strong hydrophobic interactions. Subsequently, a softer layer of Alb molecules bound together develops. Conversely, on the UVO-treated surface, the curve transitions from a steep to a gentle slope, indicating that the initial Alb adsorption produces a softer layer, with hydration water gradually released and Alb denaturing over time.

**Figure 10:**
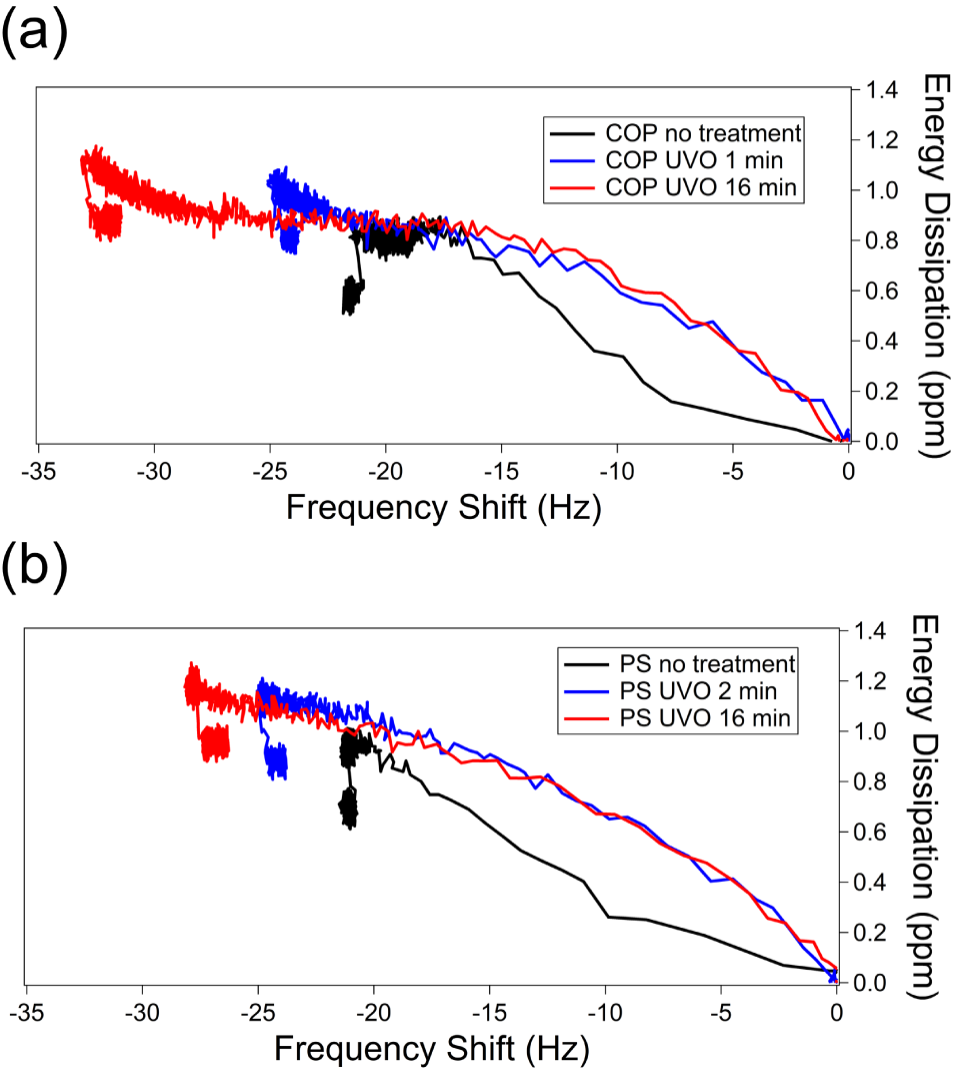
Δ*D* – Δ*f* plots showing the time progression of Alb adsorption on (a) COP and (b) PS. The fifth overtone was used in these measurements.

Protein adsorption from serum involves competitive exchange, known as the Vroman effect.^58,59^ To examine this protein exchange process, we monitored the changes in Δ*f* as a function of time during the sequential adsorption of Alb and FBS (Figure 11).

**Figure 11:**
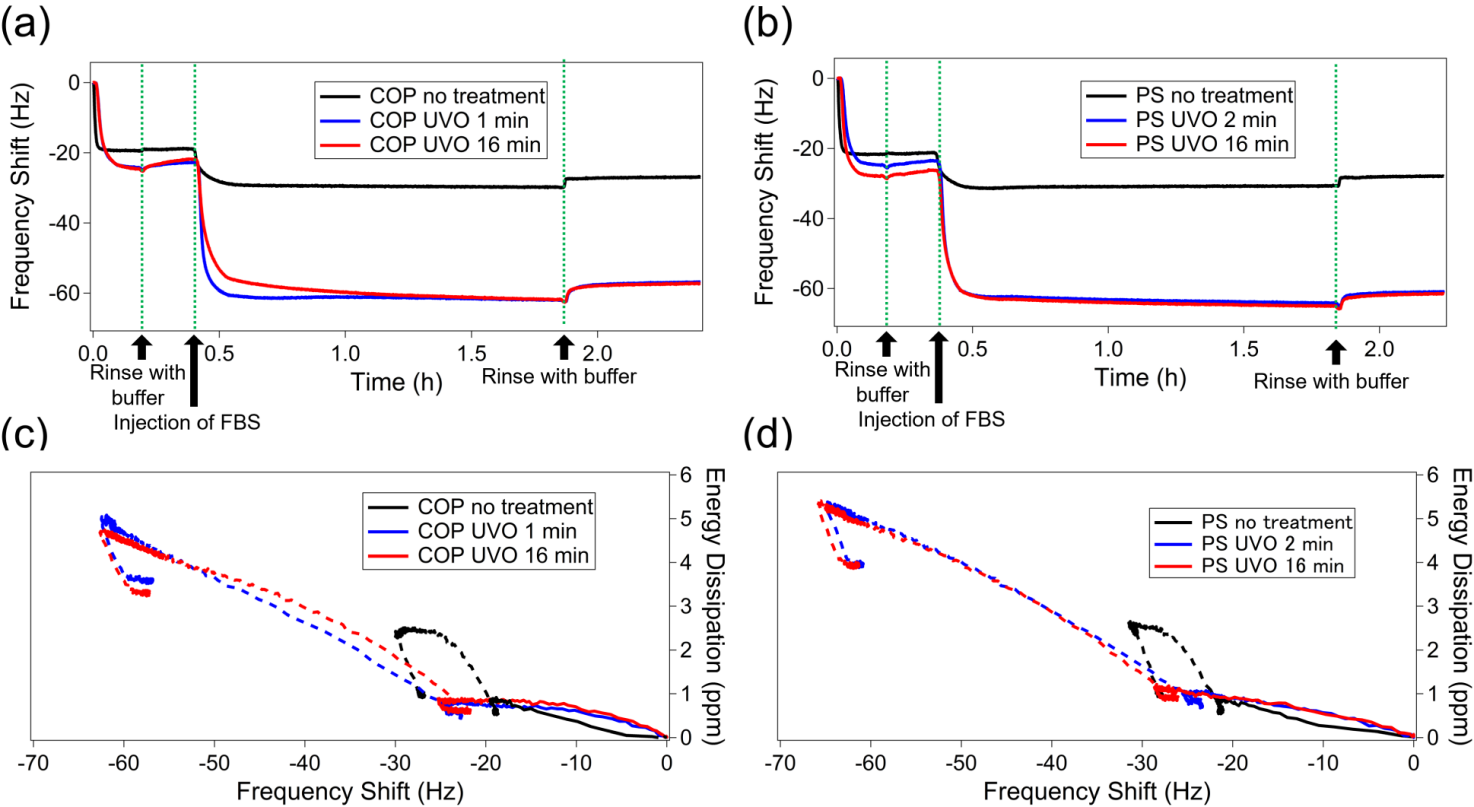
Kinetics of adsorption of serum proteins after the adsorption of Alb onto (a) COP and (b) PS at different UVO treatment durations. Δ*D* – Δ*f* plots showing the time progression of Alb and FBS adsorption on (c) COP and (d) PS. Dotted lines in (c) and (d) represent plots after injection of FBS. The fifth overtone was used in these measurements.

The Δ*f* after FBS injection on the untreated surface is lower than on the UVO-treated surface, indicating less protein exchange on untreated PS and COP surfaces compared to treated ones. Additionally, during the sequential protein adsorption process (Alb followed by FN), we observed that Alb molecules on treated polymer surfaces were displaced by FN, while those on untreated surfaces remained unchanged (Figure S1). This suggests that Alb adsorbed on hydrophobic surfaces is less likely to be replaced by other proteins, likely due to differences in the stiffness of the adsorbed layer. Multiple studies also support that Alb molecules strongly adhere to hydrophobic surfaces and resist exchange, whereas Alb weakly adsorbed on hydrophilic surfaces can be replaced by other proteins.^45,60–62^

Δ*D* – Δ*f* plots for adsorption on untreated surfaces shift from a gentle slope to a steeper one during Alb adsorption. This reflects the formation of adsorption layers, where initially strong protein attachment occurs at the material-protein interface via hydrophobic interactions, followed by a weaker layer formed through intermolecular binding. When FBS is injected later, the curve changes from steep—indicating higher energy dissipation—to shallower, showing Alb and serum proteins form a soft, weakly bound layer that denatures over time. Conversely, on the UVO-treated surface, loosely adsorbed Alb is replaced by serum proteins. The Δ*D*-Δ*f* plot for this process exhibits a gradual slope, indicative of a strong adsorption layer and high affinity between the serum proteins and the surface.

### Discussion on the Enhancement of Cell Adhesion by UVO Treatments and Its Optimization

We examine how brief UVO treatments (1 to 2 min) enhance cell adhesion. Without UVO, Alb from the culture medium easily adsorbs onto the hydrophobic surfaces of COP and PS, forming a stable, rigid protein layer confirmed by QCM-D (Figure 10). This layer results from strong hydrophobic interactions and hampers the exchange of other proteins at the interface (QCM-D, Figure 11). Alb’s surface is rich in zwitterionic amino acid pairs (e.g., EK, DK), which are known to inhibit nonspecific protein and cell adhesion. This passivating property explains why Alb is frequently used as a blocking agent in biosensing and accounts for the low protein adsorption and poor cell adhesion on untreated surfaces.

A brief UVO treatment introduces oxygen-containing functional groups onto the surfaces of COP and PS, while retaining many of the original hydrophobic regions (XPS, Tables 1 and 2). This results in a surface that is both hydrophilic and hydrophobic. The hydrophilic groups aid in the initial protein adsorption but, due to weaker interactions, also allow for their exchange (QCM-D, Figure 11). Importantly, the remaining hydrophobic areas can capture thermodynamically less stable proteins like FN and VN. When interacting with these patches, FN and VN undergo conformational shifts that reveal hidden hydrophobic residues, leading to their strong and irreversible adsorption (ELISA, Figure 6). The immobilized FN and VN then provide essential binding sites, such as RGD sequences, for cellular integrins, thereby enhancing cell adhesion.

**Figure 12:**
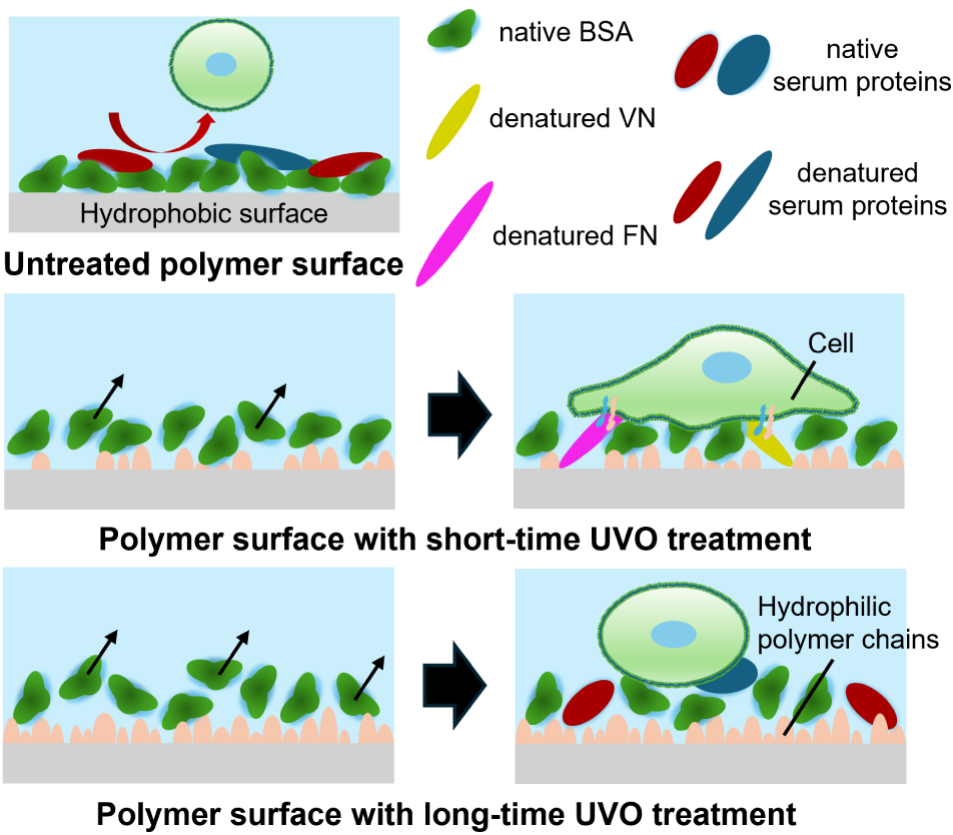
Illustration of absorbed proteins on the polymer surfaces. On untreated surfaces, Alb adsorbs rigidly to the hydrophobic surface, preventing protein exchange with adhesion proteins. On polymer surfaces with short-time UVO treatment, Alb is exchanged for FN and VN, which provides cell recognition sites and maximize cell adhesion density. On polymer surfaces with long-time UVO treatment, Alb is more likely to be exchanged for serum proteins other than FN and VN.

In contrast, a 16 min UVO treatment significantly increases the density of hydrophilic groups, rendering the surface highly water-attracting (XPS, Tables 1 and 2; WCA, 3). Such surfaces facilitate a competitive displacement process: initially adsorbed proteins are readily replaced by others with a higher affinity for the hydrophilic surface. This Vroman-like effect ultimately lowers the surface levels of FN and VN. The decrease in these key adhesion proteins, and consequently fewer RGD sites, accounts for the reduced cell density and increased circularity (less spreading) of the adhered cells.

Taken together, these results demonstrate that the optimal surface for maximizing cell density is not the most hydrophilic one. Instead, it is a moderately hydrophilized surface that retains sufficient hydrophobic domains to specifically capture and immobilize key adhesion proteins like FN and VN (Figure 6). Based on the results, we propose the detailed models of the interface as shown in Figure 12.

## Conclusion

This paper explores how UVO treatments enhance integrin-initiated adhesion of fibroblast cells on COP and PS substrates. The cell adhesion process involves several steps: hydration of the polymer surfaces, initial protein adsorption (mainly Alb), the replacement of these proteins through the Vroman effect, molecular recognition of integrin with RGD sites on VN and FN, and the formation of focal adhesions. Although many studies have aimed to understand this enhancement mechanism, a complete picture remains elusive due to the lack of comprehensive analysis of these processes.

We examined how UVO treatment affects these processes. The treatment creates oxygen-containing functional groups (carboxyl and carbonyl) on the surfaces of polymers like COP and PS, as shown by XPS analysis. It also significantly roughens the surfaces, forming protrusions observed with AFM. These surface charges from the functional groups enhance protein adsorption, which was confirmed by QCM-D measurements. The levels of VN and FN, which contain RGD motifs and support cell adhesion, peak after 2 min of UVO treatment on PS (and 1 min on COP). We developed a model to explain the protein condensation process, focusing on the interaction strength between the polymer surface and the initially adsorbed proteins, mainly Alb, at the polymer-media interface (QCM-D).

Our interfacial analysis provided the rationale model to explain the increase in adhered cells and changes in cell morphology. Fortunately, we understand ligand–receptor interactions that govern various cell behaviors such as proliferation, migration, apoptosis, and differentiation. We expect that the mechanisms triggering these cell responses can be clarified quantitatively. In this study, we used fibroblasts, whose adhesion is mainly mediated by RGD–integrin interactions, and cultured them in medium supplemented with FBS. We recognize that cell adhesion and behavior can vary depending on the cell type and culture conditions. Therefore, it will be important in future work to examine other RGD-dependent as well as RGD-independent cell types under different culture conditions to further validate our findings.

This study thoroughly examined how UVO treatment affects polymer surfaces, focusing on their physicochemical properties, serum protein adsorption, and cell adhesion. We found that UVO modifies the surface’s chemical makeup, which in turn alters the composition of adsorbed serum proteins. Short-term UVO treatment creates a protein layer that enhances cell adhesion. Our results suggest that UVO treatment of polymer surfaces can be instrumental in designing tissue culture devices, modifying polymer properties, and understanding interactions among materials, proteins, and cells.

## Supplementary Material



## Notes

The changes in surface area resulting from the formation of nanostructures due to UVO treatment of polymer surfaces and sequential protein adsorption of Alb and FN (PDF)
