# Supplementary material for: UV/Ozone Treatment of Polymer Surfaces to Enhance Cell Adhesion: The Mechanism and Guidelines for Optimization
Source: Langmuir. 2025 Oct 22;41(43):29098–110. doi: 10.1021/acs.langmuir.5c03398 (PMC12593388; doi:10.1021/acs.langmuir.5c03398)
Supplement: Supplementary file 1 [file la5c03398_si_001.pdf]

Supporting information

UV/ozone treatment of polymer surfaces to enhance cell-adhesion: the mechanism and guidelines for optimization

Riko Kaizu[1], Seiichiro Takahashi[2], Kenichi Hirose[2], Kenji Hatakeyama[2], Glenn Villena Latag[1], Ayano Nomura[1], Hiroyuki Tahara[1], and Tomohiro Hayashi[1,]*

[1] *Department of Materials Science and Engineering, School of Materials and Chemical Technology, Institute of Science Tokyo, Yokohama, 226-8502, Japan*

[2]*Ushio Inc., 6409 Motoishikawa-cho, Aoba-ku, Yokohama-shi, Kanagawa-ken, 225-0004, Japan*

*Corresponding author: th@mct.isct.ac.jp

Number of pages: 3

Number of tables: 1

Number of figures: 1

Table of Contents

Number	Title	Page
**Table S1**	Changes in surface area due to UVO treatment of polymer surfaces	S2
**Figure S1**	Kinetics of adsorption of FN after the adsorption of Alb	S3

S1 Changes in surface area due to UVO treatment of polymer surfaces

The surface area was calculated from the AFM images (5 × 5 μm²) obtained in PBS.

Table S1. Surface area of COP and PS

	Surface area (μm^2)
COP no treatment	25.0
COP UVO 1 min	25.2
COP UVO 16 min	25.5
PS no treatment	25.0
PS UVO 2 min	25.3
PS UVO 16 min	25.5

As discussed in this paper, the changes in surface area resulting from the formation of nanostructures due to UVO treatment of the polymer surfaces in this study were very small, reflecting only a marginal increase of a few percent.

S2 Sequential protein adsorption of albumin (Alb) and fibronectin (FN)

We monitored the changes in Δf as a function of time in the process of the sequential protein adsorption of Alb and FN (Fig. S1).

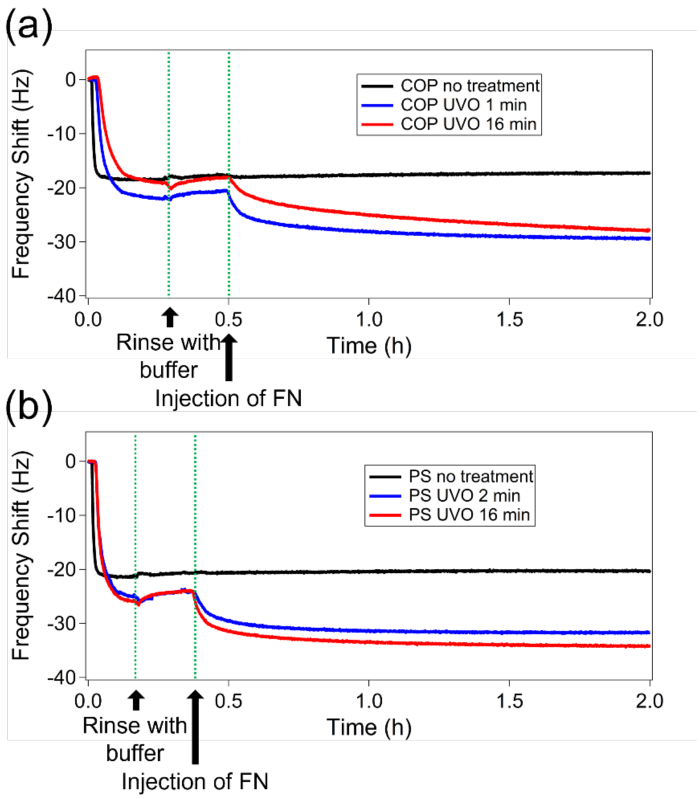

Figure S1. Kinetics of adsorption of FN after the adsorption of Alb onto (a) COP and (b) PS at different UV irradiation time. The fifth overtone was used in these measurements.

Although a negative shift in Δf due to the exchange of Alb with FN was observed at the timing of the FN injection for the UVO-treated polymer surface, no negative shift was observed for the untreated polymer surface. This indicates that the adsorbed Alb molecules on the treated polymer surfaces was displaced by FN but those on the untreated surfaces were not.
